# *Verticillium dahliae* Secretes Small RNA to Target Host *MIR157d* and Retard Plant Floral Transition During Infection

**DOI:** 10.3389/fpls.2022.847086

**Published:** 2022-04-18

**Authors:** Bo-Sen Zhang, Ying-Chao Li, Hui-Shan Guo, Jian-Hua Zhao

**Affiliations:** ^1^State Key Laboratory of Plant Genomics, Institute of Microbiology, Chinese Academy of Sciences, Beijing, China; ^2^CAS Center for Excellence in Biotic Interactions, University of Chinese Academy of Sciences, Beijing, China; ^3^School of Life Sciences, Hebei University, Baoding, China

**Keywords:** trans-kingdom RNAi, *V. dahliae*, *miR157d*, floral transition, sRNA

## Abstract

Bidirectional trans-kingdom RNA silencing [or RNA interference (RNAi)] plays a key role in plant-pathogen interactions. It has been shown that plant hosts export specific endogenous miRNAs into pathogens to inhibit their virulence, whereas pathogens deliver small RNAs (sRNAs) into plant cells to disturb host immunity. Here, we report a trans-kingdom fungal sRNA retarding host plant floral transition by targeting a miRNA precursor. From *Arabidopsis* plants infected with *Verticillium dahliae*, a soil-borne hemibiotrophic pathogenic fungus that causes wilt diseases in a wide range of plant hosts, we obtained a number of possible trans-kingdom *V. dahliae* sRNAs (VdsRNAs) by sequencing AGO1-immunoprecipitated sRNAs. Among these, a 24-nt VdsRNA derived from *V. dahliae* rRNA, VdrsR-1, was shown to be an actual trans-kingdom VdsRNA that targets the miR157d precursor *MIR157d*, resulting in increased rather than reduced miR157d accumulation in *V. dahliae*-infected plants. Consistent with the miR157 family in the regulation of vegetative and floral transitions by targeting *SPL* genes in several plant species, we detected two *SPL* genes, *SPL13A/B*, that were notably reduced in *V. dahliae*-infected and VdrsR-1-expressing plants compared with control plants. Furthermore, *V. dahliae*-infected and VdrsR-1-expressing plants also displayed delayed vegetative phase change and floral transition compared to control plants. Taken together, we disclosed a novel mode of action for a trans-kingdom fungal sRNA, VdrsR-1, which was secreted into host cells to modulate plant floral transition by employing the miR157d/*SPL13A/B* regulatory module, leading to prolonged host vegetative growth that would undoubtedly benefit fungal propagation.

## Introduction

In most eukaryotes, RNA silencing [or RNA interference (RNAi)] is crucial for normal development and defense against biotic and abiotic stress. Small RNAs (sRNAs), as the key mediators of RNAi, are divided into microRNAs (miRNAs) and small interfering RNAs (siRNAs) according to their origin (Treiber et al., 2019; Wang et al., 2019; Chen and Rechavi, 2021). Generally, miRNAs are derived from primary miRNA transcripts (pri-miRNAs) containing an imperfect hairpin structure (precursor, pre-miRNA) that are sequentially processed by the RNase III enzyme Dicers (Moran et al., 2017). In *Arabidopsis thaliana*, one of the Dicer homologous proteins Dicer-like 1 (DCL1) is the primary enzyme involved in miRNA biogenesis, which processes pri-miRNAs into pre-miRNA and ∼20–24-nt miRNA/miRNA* duplexes in two steps (Song et al., 2019). Mature miRNAs load onto the AGO1 protein to form miRNA-induced silencing complexes (miRISCs). On the basis of sequence complementarity, miRISC negatively regulates gene expression by directing target mRNA degradation or translational inhibition (Yu et al., 2017; Song et al., 2019; Wang et al., 2019).

Massive evidence indicates that miRNAs are key regulators of plant development. The miR156/157 family is one of the most conserved miRNA families in all land plant lineages (Axtell and Bowman, 2008; Liu et al., 2017) and coordinates vegetative and floral transitions by targeting *SPL* genes in *Arabidopsis* (Schwab et al., 2005; Wu and Poethig, 2006; Wang et al., 2008; Xu et al., 2016; He J. et al., 2018), maize (Chuck et al., 2007), cotton (Liu et al., 2017; He X. et al., 2018), and several other species (Shikata et al., 2012; Gao et al., 2018). A series of miRNAs regulate plant development by targeting genes involved in hormone biosynthesis and signaling, such as miR164:*NAC1* (Guo et al., 2005), miR160/167:*ARF*s (Mallory et al., 2005; Wu et al., 2006; Liu et al., 2007), miR159:*MYB*s (Millar and Gubler, 2005; Reyes and Chua, 2007), and miR847:*IAA28* (Wang and Guo, 2015). In addition, miRNA-mediated regulation of gene expression plays an important role in the plant response to abiotic stresses. In rice, miR528 enhances plant resistance to viruses by increasing the production of reactive oxygen species (Wu et al., 2015, 2017). A recent study showed that *Brassica* miR1885 dynamically regulates both innate immunity and plant growth and responds to viral infection through distinct modes of action (Cui et al., 2020). The vital roles of miRNAs in the regulation of plant development, phenotypic plasticity, abiotic and biotic responses, as well as symbiotic and parasitic interactions have been summarized in several excellent reviews (Jones-Rhoades et al., 2006; Chen, 2009; D’Ario et al., 2017; Tang and Chu, 2017; Huang et al., 2019; Song et al., 2019, 2021; Wang et al., 2019; Dexheimer and Cochella, 2020; Liu et al., 2020; Chen and Rechavi, 2021; Qiao et al., 2021).

Bidirectional trans-kingdom RNAi has been demonstrated to influence plant host-pathogen interactions. An early study reported that *Botrytis cinerea* sRNAs are transmitted into hosts during infection, functioning as RNA effectors to perturb plant immune signaling pathways (Weiberg et al., 2013). In this study, Bc-siR3.2 hijacked the host RNAi machinery by loading into AGO1 to target plant mitogen-activated protein kinase transcripts, thereby suppressing host immunity to facilitate infection (Weiberg et al., 2013). More recently, a study demonstrated that *Fol-milR1*, a pathogenicity factor of *Fusarium oxysporum*, degrades the tomato *SlyFRG4* gene, which is essential for tomato wilt disease resistance by binding to the tomato SlyAGO4a protein (Ji et al., 2021). In oomycetes, the sRNAs of *Hyaloperonospora arabidopsidis* were reported to employ the host AGO for virulence (Dunker et al., 2020). On the other hand, hosts export specific endogenous miRNAs into pathogens to confer host disease resistance by targeting pathogen virulence genes (Hua et al., 2018; Zhao and Guo, 2019; Zhao et al., 2021). In our previous study, we reported that cotton plants export conserved miRNAs into the pathogenic fungus *Verticillium dahliae* (V592 strain) to inhibit fungal virulence genes. We identified 28 different cotton miRNAs from *V. dahliae* recovered from infected cotton plants. Further analysis demonstrated that miR166 and miR159 cleave the transcripts of the *Clp-1* and *HiC-15* genes, which are essential for hyphal growth and microsclerotium formation, respectively (Zhang et al., 2016). Recently, *V. dahliae* miRNA-like RNAs (VdmilRNAs) have also been identified, and VdmilR-1 represses fungal endogenous target gene expression at the transcriptional level by increasing histone H3K9 methylation (Jin et al., 2019). However, whether *V. dahliae* secretes sRNAs to regulate host genes has rarely been reported.

In the present study, aiming to identify and uncover the functions of *V. dahliae* sRNA (VdsRNA) classes associated with host AGO1 protein during fungal infection, we immunoprecipitated AGO1 using the c-myc antibody from 6myc-AGO1-overexpressing *Arabidopsis* plants (6myc-AGO1) with or without *V. dahliae* infection. Total RNA was extracted from the AGO1-IP fraction, and sRNA libraries were constructed. By analyzing sequencing data, we identified that an AGO1-associated VdsRNA derived from *V. dahliae* rRNA, named VdrsR-1, targets the precursor of host miR157d, *MIR157d*. Unexpectedly, rather than reducing miR157d accumulation, VdrsR-1 increased miR157d accumulation in the *V. dahliae*-infected plants. Consistently, the accumulation levels of two *SPL* genes, *SPL13A/B*, predicted targets of miR157d, were notably reduced in *V. dahliae*-infected plants. Phenotypic resemblance between *V. dahliae*-infected plants and VdrsR-1-overexpressing plants suggests that the trans-kingdom VdrsR-1 plays a role in delaying host floral transition by exploiting the miR157d*/SPL13A/B* module, probably beneficial to fungal development inside the infected plants.

## Materials and Methods

### Plant Materials and Manipulations

6myc-AGO1 *Arabidopsis* plants (Col-0 background) were obtained by transforming 35S-6myc-AGO1 into the *ago1-27 Arabidopsis* mutant (Duan et al., 2012). *Arabidopsis* plants were grown in soil in a greenhouse at 22°C under long-day conditions (16 h/8 h day/night) with 60% humidity. For fungal infection, 10-day-old seedling mutants were uprooted, and the roots were dipped for 5 min in 1 × 10^7^ cfu/ml spores of *V. dahliae* (V592 strain). After inoculation, the plants were transferred to soil. Control plants were treated similarly with water. Samples were collected at 2 weeks postinoculation for RNA extraction and immunoprecipitation. The pathogenic phenotype of *Arabidopsis* was recorded at 2 and 4 weeks during the experimental period. All of the infection assays were repeated three times. For transient expression, *N. benthamiana* plants were grown in soil under long-day conditions at 25°C for 4 weeks.

### Vector Construction

In this article, all of the constructs were ligated by In-fusion cloning methods using the ClonExpress II One Step cloning kit (Vazyme, Nanjing, China).

For the *35S-MIR157d* and *35S-MIR159a* constructs, the 221-bp *MIR157d* and 184-bp *MIR159a* gene sequences were amplified by RT-PCR and ligated into the *Xba*I*-Sac*I-linearized pCAMBIA1300-221 binary vector. To generate artificial miRNA precursor skeletons used in infiltration assays, we synthesized all of the precursors by GenScript (Nanjing, China). We used precursor *MIR5653* to express 24-nt VdrsR-1 and amiR_*t*159_, respectively. The 86-bp sequence was amplified by PCR and ligated into *Xba*I*-Sac*I-linearized pCAMBIA1300-221 binary vector. For the *35S-MIR157dm* and *35S-MIR157d_*asR–*1_* constructs, the 221-bp sequences were amplified by PCR and ligated into the *Xba*I-*Sac*I-linearized pCAMBIA1300-221 binary vector. For the *35S:SPL13B* construct, the 1817-bp *SPL13B* gene sequence, which is consistent with partial sequence of *SPL13A*, was amplified by RT-PCR and ligated into the *Xba*I*-Sac*I-linearized pCAMBIA1300-221 binary vector. To generate TRV-VdsrR-1, the 86-bp sequence was amplified and fused into the pTRV2 vector according to a previous report (Liu et al., 2002). All primers are listed in Supplementary Table 1.

### RNA Isolation and RT-qPCR Analysis

*Arabidopsis* tissue and *N. benthamiana* leaves were collected for total RNA, using TRIzol reagent (Invitrogen, Carlsbad, CA, United States). Genomic DNA removal and reverse transcription were performed using the HiScript III RT SuperMix for qPCR kit (Vazyme, Nanjing, China). RT-qPCR was analyzed by a CFX96 real-time system (Bio-Rad, Hercules, CA, United States) using SYBR Green PCR master mix (Vazyme, Nanjing, China). The primers used in RT-qPCR were listed in Supplementary Table 1.

### AGO Protein Immunoprecipitation

*Arabidopsis* protein was extracted from 8 g fresh plants collected at 2 weeks postinoculation with V592 and the control. AGO1 protein was purified with prepared c-MYC Dynabeads. For the negative control, another anti-strep II bead was incubated in a parallel process.

### Small RNA Sequencing and Analysis

After total RNA was extracted from the AGO1-IP fraction, sRNA library construction and sRNA sequencing were performed with Illumina HiSeq™ 2500 by Gene Denovo Biotechnology Co. (Guangzhou, China)^1^. Three repeat libraries were constructed. Clean reads were aligned to the *Arabidopsis* reference genome (TAIR10). By using read counts >10 in all three repeats as a cutoff, the unmatched reads were aligned to the *V. dahliae* genome (assembly ASM15067v2). Known *Arabidopsis* miRNAs were downloaded from miRBase release 22 (Griffiths-Jones et al., 2008). sRNAs with lengths between 18 and 30 nt were included in our analysis, and sRNA abundance was normalized into reads per million (rpm). psRNATarget (Dai et al., 2018) with a maximum expectation score of 3 was used to predict the targets of VdsRNAs. The function of VdsRNA targets was analyzed by AgriGO (Du et al., 2010), and enriched gene ontology (GO) terms were visualized by WEGO (Ye et al., 2018).

### Small RNA RT-PCR

RNA was extracted from *Arabidopsis* or the 6myc-AGO1 plant tissue-bound RNA fraction using TRIzol reagent (Invitrogen, Carlsbad, CA, United States). AGO1-sRNA reverse transcription was performed using the miRNA 1st Strand cDNA Synthesis Kit (Vazyme, Nanjing, China). 28 cycles were used for detecting VdrsR-1. For the positive and negative controls, 35 cycles were used to detect miR162, miR168, siRNA1003 and VdmilR-1. The primers used in sRNA RT-PCR are listed in Supplementary Table 1.

### *N. benthamiana* Agrobacterium Infiltration Assays

Agrobacterium infiltration assays were performed according to a previously described method (Duan et al., 2012). With the method of electroporation, constructs were transformed into *Agrobacterium* strain EHA105 Competent individually. A single colony was cultured overnight in 5 ml LB selection medium. 1 ml of LB medium was transferred into 20 ml of LB medium and grown for 16 h. The next day, bacterial culture media were harvested and resuspended in 10 mM MgCl_2_ buffer at an optical density before infiltration. *N. benthamiana* leaves were collected at 4 days for RNA extraction.

### *Arabidopsis* Virus-Induced Gene Silencing Assays

*Agrobacterium* (EHA105) was used to transform pTRV1 or pTRV2 and its derivative constructs into *Arabidopsis.* The Agrobacterium transformation and infiltration methods were consistent with those previously described (Duan et al., 2012). 14 days, *Arabidopsis* was treated with shading overnight before and after infiltration. The plant tissues were collected at 14 days post-infiltration for RNA extraction.

### Plant RNA Gel Blot Assays

Plant total RNA and sRNA gel blot methods were described in a previous report (Duan et al., 2012). For detection of *DCL1* and *SPL13A/B*, DNA probes were amplified by PCR and then labeled with [α-^32^P]dCTP with the Rediprime II system (Amersham, Buckinghamshire, United Kingdom). For the detection of specific sRNAs, probes were labeled with T4 polynucleotide kinase (NEB, Beijing, China) with [γ-^32^P]ATP. The primers and probes used in the RNA gel blot are listed in Supplementary Table 1.

## Results

### Identification of *Arabidopsis* AGO1-Associated Trans-Kingdom *V. dahliae* sRNAs

**FIGURE 1:**
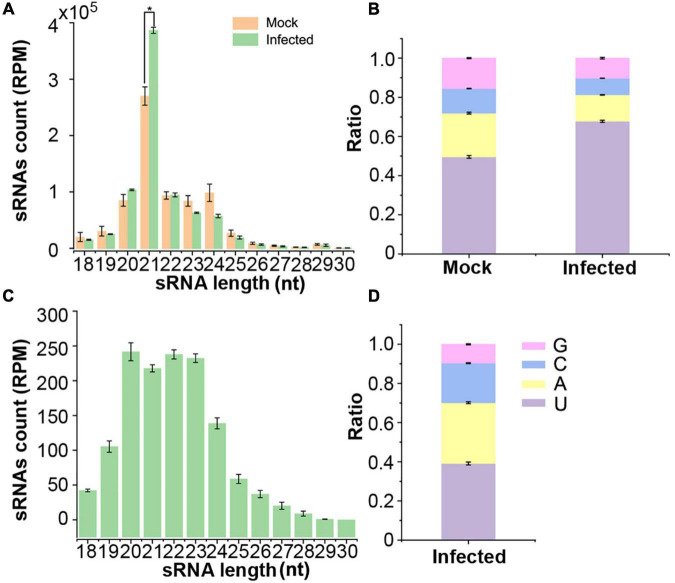
Deep sequencing profiling of *Arabidopsis* AGO1-associated sRNAs in mock and *V. dahliae*-infected plants. **(A)** Numbers of AGO1-immunoprecipitated (AGO1-IPed) sRNAs of different lengths from plants with or without *V. dahliae* infection. **(B)** The 5′ terminal nucleotide compositions of AGO1-IPed sRNAs in different libraries. **(C)** The numbers of different lengths of AGO1-IPed sRNAs aligned to the *V. dahliae* genome. **(D)** The 5′ terminal nucleotide compositions of AGO1-IPed sRNAs aligned to the *V. dahliae* genome. Values are the means ± SD, and asterisk indicates statistically significant differences (*n* = 3; *t*-test, *P* < 0.05).

To identify VdsRNAs that might be transferred into host plant cells and functionally, 6myc-AGO1 transgenic *Arabidopsis* plants in which the *ago1* mutation was complemented by a myc-tagged AGO1 construct (Duan et al., 2012) were used for infection with *V. dahliae*. Anti-α-myc antibody was then used to immunoprecipitate AGO1 from *V. dahliae*-infected and mock-inoculated 6myc-AGO1 plants at 2 weeks postinfection. AGO1-IP sRNAs were isolated and sequenced. Three replicates for each sample were carried out. After removing low-quality reads, we obtained approximately 20 M reads for each library. The AGO1-IP sRNAs from mock plants were dominated by 21-nt classes and 5′-terminal uracil (1U, 50%) (Figures 1A,B), which were consistent with previous studies (Mi et al., 2008; Wang et al., 2011). Similarly, the AGO1-IP sRNAs from *V. dahliae*-infected plants were also mainly 21 nt long with 5′-terminal uracil (Figures 1A,B). However, AGO1-IP 21-nt sRNAs were much more abundant, and the ratio of sRNAs with 1U (68%) was increased in *V. dahliae*-infected plants (Figures 1A,B). After filtering out sRNAs mapped to the *Arabidopsis* genome, the remaining reads that were mapped to the *V. dahliae* genome from AGO1-IP sRNAs in *V. dahliae*-infected plants were assumed to be “trans-kingdom” VdsRNAs. These AGO1-IP “trans-kingdom” VdsRNAs were mainly 20–24 nt in length, with high proportions of 1A (31%) and 1U (39%) (Figures 1C,D).

**FIGURE 2:**
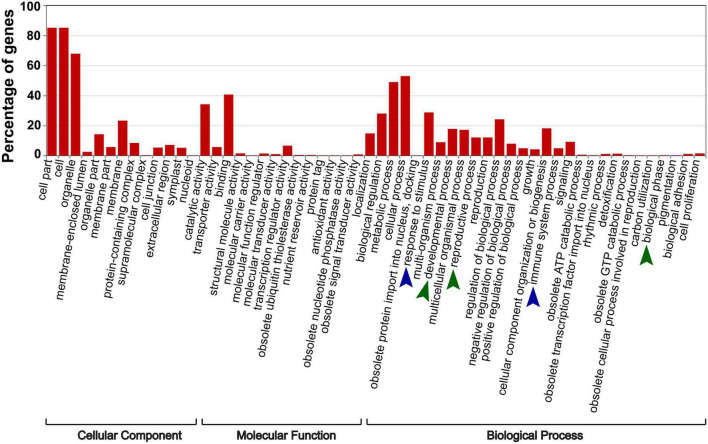
The specific enriched GO terms of putative target genes of AGO1-IPed VdsRNAs. 1269 *Arabidopsis* mRNAs were predicted as potential targets of these VdsRNAs. Blue arrowheads mark the enriched GO terms involved in host immunity, and green arrowheads mark the enriched GO terms involved in plant development.

To investigate whether the “trans-kingdom” VdsRNAs possessed possible biological functions, we focused on 705 unique “trans-kingdom” VdsRNAs by using read counts >10 in all three repeats as a cutoff. Next, 1269 *Arabidopsis* mRNAs were predicted as potential targets of these “trans-kingdom” VdsRNAs. GO annotation results showed that these target genes were categorized into extensive pathways (Figure 2). Unsurprisingly, dozens of potential target genes were involved in immune system processes and responses to stimuli (Figure 2), in agreement with previous reports of trans-kingdom sRNAs acting as RNA effectors to suppress host immunity (Weiberg et al., 2013; Dunker et al., 2020; Ji et al., 2021). In addition, we noted that several target genes were annotated as developmental process, reproductive process or biological phase (Figure 2). Thus, our data suggest that these “trans-kingdom” VdsRNAs probably facilitate *V. dahliae* infection and regulate plant development by hijacking the host AGO1 protein.

### Confirmation of a 24-nt Fungal rRNA-Derived *V. dahliae* sRNA Loaded Into Host AGO1

**FIGURE 3:**
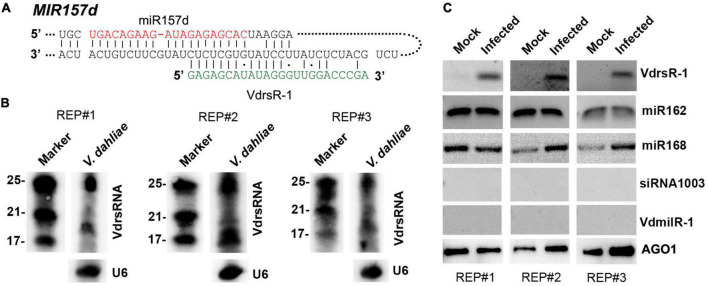
Confirmation of the 24-nt *V. dahliae* rRNA-derived VdsRNA (VdrsR-1) loaded into host AGO1 protein. **(A)** The schematic diagram shows the complementarity between VdrsR-1 and *MIR157d*. The mature miR157d and VdrsR-1 sequences are shown in red and green letters, respectively. **(B)** Northern blotting results with three biological replicates showed that *V. dahliae* generated abundant rRNA-derived ∼24-nt and smaller sRNAs. U6 served as loading control. **(C)** VdrsR-1 was detected by RT-PCR in AGO1-IPed sRNA from *V. dahliae*-infected plants. Two known AGO1-loaded miR162 and miR168 were used as positive controls. A plant endogenous 24-nt siRNA1003 and a *V. dahliae* miRNA-like VdmilR-1 that were not present in AGO1-IPed sequencing data were also examined and not detected. Anti-α-myc antibody was used to detect the amount of AGO1-IPed from mock and infected plants. The results of three independent replicates are shown.

Among the putative “trans-kingdom” VdsRNA targets that potentially regulated plant development, we noticed that the plant miR157d transcript matched a 24-nt trans-kingdom VdsRNA derived from *V. dahliae* rRNA (Figure 3A). It is well known that miRNAs of the miR156/157 family coordinate vegetative and floral transitions by regulating *SPL* genes in several plant species. This prompted us to investigate whether the 24-nt VdsRNA was indeed derived into plant cells and loaded into host AGO1 as a bona fide trans-kingdom VdsRNA, and the miR157d gene was a genuine target of this trans-kingdom VdsRNA.

We first investigated whether *V. dahliae* rRNA produced sRNAs in fungal cells. Northern blot analysis was performed to detect sRNAs in *V. dahliae* cultured on PDA plates using a 24-nt VdsRNA-specific oligo probe. The hybridization results showed that *V. dahliae* rRNA generated abundant ∼24-nt VdsRNAs. Interestingly, ∼20-nt or even smaller bands with strong hybridization signals were also detected (Figure 3B). However, these ∼20-nt and smaller VdsRNAs were not present in the AGO1-IP sRNA sequencing data, even though a large number of other 20-nt and smaller VdsRNAs were in the AGO-IP samples (Figure 1C). These data suggest that there was possibly selectivity for either the delivery of VdsRNAs into plant cells or the loading of VdsRNAs into AGO1. Together with the *V. dahliae* miRNA-like sRNA, VdmilR-1, which has previously been reported to regulate the fungal endogenous gene at the transcriptional level (Jin et al., 2019), was not present in any AGO1-IP VdsRNA libraries. We reasoned that the AGO1-IP VdsRNAs, particularly the 24-nt tested VdsRNAs, were not due to their high accumulation in fungal cells and contamination during AGO1-IP manipulation. Hereafter, this 24-nt VdsRNA derived from *V. dahliae* rRNA was named VdrsR-1 in this study.

Next, we further confirmed the AGO1-associated VdrsR-1. RT-PCR assays were performed, and the results showed that VdrsR-1 was detected in the three replicates of AGO1-IP sRNAs from *V. dahliae*-infected plants but was absent in the samples from mock plants (Figure 3C). *Arabidopsis* miR162 and miR168, which have been reported to be loaded into AGO1, were also detected in AGO1-IP sRNAs from plants with or without *V. dahliae* infection (Figure 3C). *Arabidopsis* AGO4-associated siRNA1003 was used as a negative control and was not detected in any AGO1-IP samples (Figure 3C). Moreover, VdmilR-1, which was not present in the AGO1-IP VdsRNA libraries, was not detected in any samples (Figure 3C). Taken together, our data demonstrate that *V. dahliae* produces abundant 24-nt VdrsR-1 VdsRNAs that are secreted into plant host cells during infection and that VdrsR-1 is a bona fide trans-kingdom VdsRNA loaded into the host AGO1 protein.

### *V. dahliae* Infection Increased the Accumulation of miR157d

**FIGURE 4:**
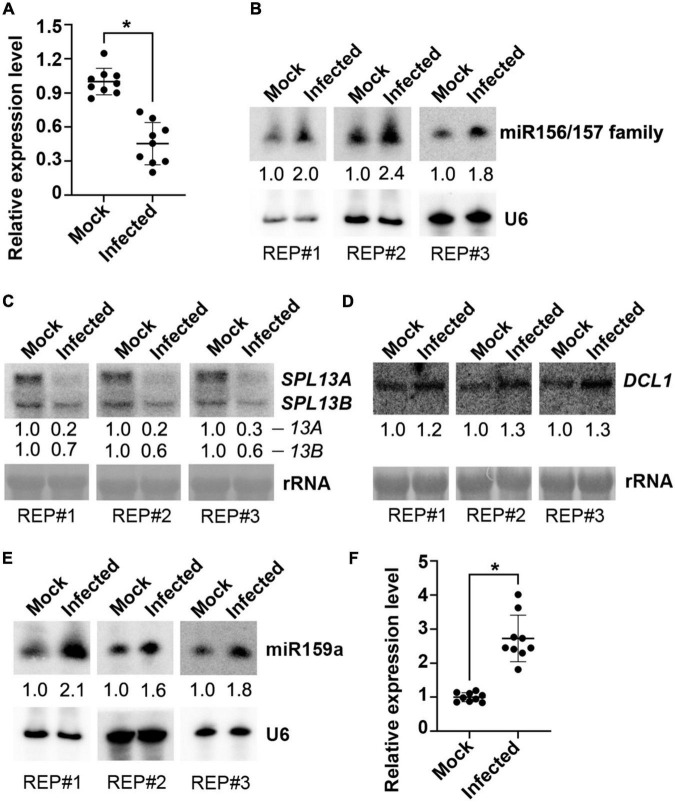
Examination of miR157d and miR159 and related genes in response to *V. dahliae* infection. **(A,F)** Detection of the transcription of *MIR157d* **(A)** and *MIR159a* **(F)** by RT-qPCR. The asterisk indicates significantly different expression of mock plants versus infected plants (*n* = 9; *t*-test, *P* < 0.05). **(B,E)** Detection of the accumulation of miR156/157 family **(B)** and miR159 **(E)** by Northern blotting. U6 hybridization was used as a loading control. **(C,D)** Detection of the expression of *SPL13A/B* genes **(C)** and *DCL1* gene **(D)** by Northern blotting. rRNA was stained with methylene blue as a loading control. The Northern blotting signals were quantified and normalized first to those of U6 or rRNA and then to mock. The results of three biological replicates are shown.

Next, we examined whether there was a change in the miR157d gene, the putative target of VdrsR-1, after *V. dahliae* infection. We first performed RT-qPCR to measure the transcript of *Arabidopsis* miR157d transcript, the precursor *MIR157d*, in *V. dahliae*-infected and mock-inoculated control plants. The level of *MIR157d* was significantly reduced in *V. dahliae*-infected plants compared with that in control plants (Figure 4A). Subsequently, we explored whether the reduced *MIR157d* would result in reduced production of mature miR157d. Unexpectedly, AGO1-IPed miR157d was slightly increased in *V. dahliae*-infected plants compared with that in control plants (Supplementary Figure 1A). We then used miR157d oligo probe to detect the accumulation level of miR157d by Northern blot analysis. The hybridization signals were obviously increased in *V. dahliae*-infected plants (Figure 4B). In view of the members of miR156/157 family miRNAs having high similarity sequences, the enhancement of hybridization signals would be due to the increased accumulation of miR156/157 family members including miR157d. In agreement with this, we found that AGO1-IPed miR157a/b/c were also slightly increased upon *V. dahliae* infection (Supplementary Figure 1A).

At the top of the overexposed Northern blotting membranes, two specific hybridization bands were also detected. Decreased upper band signals and increased lower band signals were observed in *V. dahliae*-infected plants compared with control plants (Supplementary Figure 1B). In view of the reduced *MIR157d* in *V. dahliae*-infected plants detected by RT-qPCR (Figure 4A), we inferred that the upper band was the *MIR157d* precursor and that the lower band could be the DCL1-processed miR157d-containing intermediate produced as hybridized by the miR157d-specific oligo probe. The VdrsR-1 target site at the 3′-end of the *MIR157d* sequence made it difficult to use the 5′ RACE method to examine whether VdsR-1 mediates cleavage of *MIR157d.* However, the decreased accumulation of *MIR157d* but increased the intermediate product and miR157d suggested that AGO1-associated VdrsR-1 targeting presumably promotes DCL1-mediated *MIR157d* processing to produce miR157d rather than causing degradation of *MIR157d*. We then examined the expression levels of *SPL* genes, the predicted target gene family of miR157d (Supplementary Figure 2), and found that the expression levels of *SPL13A/B* genes with highly similar sequences (Supplementary Figure 3) were obviously reduced in *V. dahliae*-infected plants compared with those in control plants (Figure 4C and Supplementary Figure 2). These data demonstrate that *V. dahliae* infection reduced the expression of *SPL13A/B* resulting from the increased accumulation of miR157d, presumably due to VdrsR-1-promoted DCL1-mediated *MIR157d* processing.

We then examined the expression of *DCL1*, which is responsible for processing pre-miRNAs into mature miRNAs in *Arabidopsis* (Song et al., 2019). We found that *DCL1* was slightly induced in *V. dahliae*-infected plants (Figure 4D). This result was consistent with our previous finding that a class of miRNAs were increased upon *V. dahliae* infection (Jin et al., 2018). Therefore, we assumed that the slight induction of *DCL1* in *V. dahliae*-infected plants, at least partly, contributed to the increase in miR157d and other endogenous miRNAs. To test whether other increased miRNAs were also accompanied by reduced precursor levels, miR159a, which has been confirmed to be induced by *V. dahliae* infection in cotton and *Arabidopsis* plants (Zhang et al., 2016; Jin et al., 2018) was selected for examination. As expected, the accumulation of miR159a was increased upon *V. dahliae* infection (Figure 4E). However, unlike the reduction in *MIR157d*, *MIR159a* was induced upon *V. dahliae* infection (Figure 4F). These data together indicated that increased accumulation of miR157d and miR159a in response to *V. dahliae* infection was through distinct modes. Although it was not clear how *V. dahliae* infection induced the expression of *DCL1* and *MIR159a* (Figures 4D,F), VdrsR-1 would play a role in promoting DCL1-mediated processing of *MIR157d*. Previous studies revealed that the secondary structure of the precursors determines their processing pathway by DCL1 (Bologna et al., 2009, 2013; Liu et al., 2012; Zhu et al., 2013). Therefore, we analyzed the possible secondary structure of *MIR157d* using mfold (Zuker, 2003). In addition to a long near-perfect hairpin structure in which the mature miR157d sequence positions at the 5’-end region, the *MIR157d* precursor contains a typical terminal loop. VdrsR-1 partially paired to the 3’-end of the upper part of the stem with the 5’-terminal 8 nucleotides overlapping with the 3’-terminal 8 nucleotide of miR157d (Supplementary Figure 4). We speculated that the unique stem-loop structure and the pairing between VdrsR-1 and *MIR157d* might lead to the outcome of AGO1-VdrsR-1-mediated action facilitating DCL1 process of *MIR157d* into mature miR157d.

### Examination of the VdrsR-1-Facilitated Procession of *MIR157d*

**FIGURE 5:**
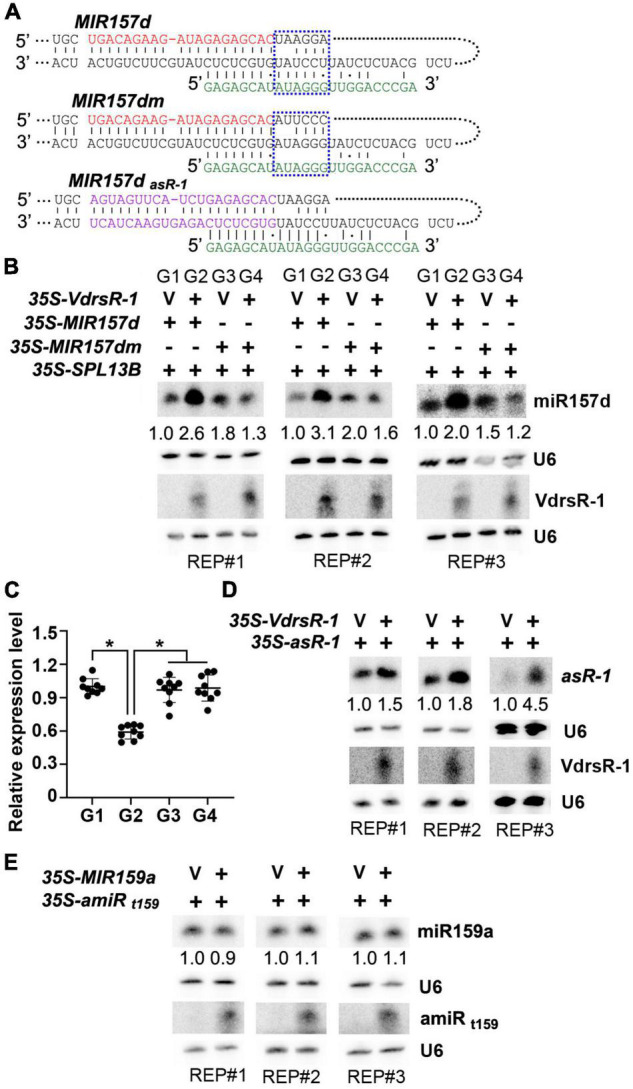
VdrsR-1 triggered miR157d accumulation and reduced *SPL* gene expression. **(A)** The schematic diagram shows the complementarities of VdrsR-1 with wild-type *MIR157d*, with mutated *MIR157dm*, and with *MIR157d_*asR–*1_*. The mature miR157d and VdrsR-1 sequences are shown in red and green letters, respectively. The blue dashed lines mark the wild-type and mutated nucleotides within the *MIR157d* and *MIR157dm* sequences. The purple letters in *MIR157d_*asR–*1_* indicate that miR157d and related complementarity nucleotides were substituted to produce art-sRNA-1. **(B)** *35S-VdrsR-1* was transiently co-expressed with *35S-MIR157d* or *35S-MIR157d* in the presence of *35S-SPL 13B* in *N. benthamiana* leaves. V indicates an empty vector. The accumulation levels of miR157d and VdrsR-1 were detected by Northern blotting. U6 hybridization served as loading control. **(C)** The relative expression level of *SPL13B*. The asterisk indicates significant difference expression of co-expression of *35S-VdrsR-1* with 3*5S-MIR157d* (G2) versus other co-expression combinations (G1, G3, and G4) (*n* = 9; *t*-test, *P* < 0.05). **(D)** *35S-VdrsR-1* was transiently co-expressed with *35S- MIR157d_*asR–*1_* in *N. benthamiana* leaves. V indicates an empty vector. The accumulation levels of asR-1 and VdrsR-1 were detected by Northern blotting. U6 hybridization served as loading controls. **(E)** Co-expression of *MIR159a* with *35S-amiR_*t*159_*, which targets *MIR159a*. The accumulation levels of miR159a and amiR_*t*159_ were detected by Northern blotting. U6 hybridization served as loading controls. The Northern blotting signals were quantified and normalized first to those of U6 and then to corresponding control. The experiments were repeated three times.

To test whether VdrsR-1 facilitated the process of *MIR157d*, several *Arabidopsis* miRNA precursors were first examined for their capacity to produce specific 24-nt artificial sRNAs and found that the miRNA precursor MIR5653 could generate better 24-nt VdrsR-1 (Supplementary Figure 5). Therefore, MIR5653 was used to construct an artificial precursor derived by the 35S promoter, *35S-VdrsR-1*, to produce 24-nt VdrsR-1. Transient expression system in *N. benthamiana* was used to co-express *35S-MIR157d* and *35S-VdrsR-1* or a vector control together with *35S-SPL13B*, one of the miR157d target genes for indication of accurate production and function of miR157d in this transient expression system. As shown in Figure 5, co-expression of *35S-VdrsR-1* significantly increased miR157d accumulation and decreased *SPL13B* mRNA compared to co-expression with a vector control. To further test the requirement of pairing with VdrsR-1 for the increased accumulation of miR157d, we mutated the *MIR157d* precursor, *MIR157dm*, in which the miR157d-containing sequence and stem-loop structure were maintained; however, six nucleotides in the upper part of stem-loop were substituted not to be matched by VdrsR-1 for the 5’-end 9–14 nucleotides (Figure 5A and Supplementary Figure 6). Increased miR157d accumulation and decreased *SPL13B* mRNA were not detected after co-expression of *MIR157dm* with *35S-VdrsR-1* compared to the vector control (Figures 5B,C). These data demonstrated that the mutant *MIR157dm* was able to produce mature miR157d and that pairing of wild-type *MIR157d* with VdrsR-1 was required for the facilitation of the *MIR157d* process.

To further examine whether the precursor structure rather than the mature miR157d sequence was essential for the VdrsR-1-facilitated DCL1 process, we used the precursor *MIR157d* to express a sequence-specific artificial sRNA, asR-1. Transient expression assays showed that co-expression of VdrsR-1 clearly increased asR-1 accumulation compared to co-expression with a vector control (Figure 5D). Additionally, we used *MIR5653* to express a 24-nt artificial miRNA, *35S-amiR_*t*159_*, to target the *MIR159a* precursor at a similar position of VdrsR-1 to *MIR157d* (Supplementary Figure 7). After co-expression of *MIR159a* with *35S-amiR_*t*159_* or a vector control, similar accumulation of miR159a was detected in either co-expression sample (Figure 5E), indicating that the *MIR159a* precursor targeted by a 24-nt amiR_*t*159_ did not facilitate the DCL1-mediated *MIR159a* process. These data demonstrated that VdrsR-1-promoted miR157d accumulation was neither due to the miR157d nucleotide sequence nor merely due to matching to a precursor sequence.

Taken together, our data demonstrated that trans-kingdom VdrsR-1-triggered miR157d accumulation resulted from AGO1-associated VdrsR-1 targeting to *MIR157d*, which bears a typical secondary structure, facilitating DCL1-mediated processing of MIR157d and truly increasing the accumulation of miR157d.

### VdrsR-1 Delayed the *Arabidopsis* Floral Transition

Previous studies reported that overexpression of miRNAs in the miR156/157 family could induce bushy architecture and delay phase transition in several plant species (Schwab et al., 2005; Chuck et al., 2007; Shikata et al., 2012; Liu et al., 2017; He X. et al., 2018). *V. dahliae* infection increased host miR157d accumulation (Figure 4B), prompting us to inspect the plant growing phenotypes with and without *V. dahliae* infection.

**FIGURE 6:**
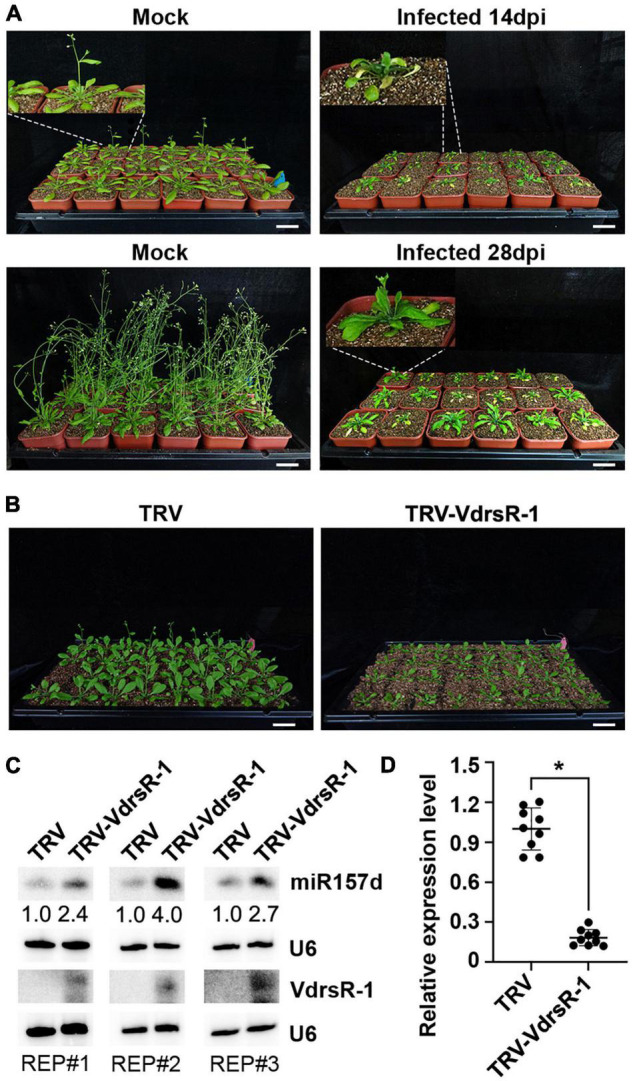
Trans-kingdom fungal VdrsR-1-promoted plant miR157d accumulation partially contributed to *V. dahliae*-induced delayed floral transition in plants. **(A)** Phenotypic comparison of *V. dahliae*-infected plants and mock-inoculated plants at 14 and 28 dpi. In general, *V. dahliae*-infected plants displayed wilting, necrosis and serious stunting, as well as delayed floral transition. Scale bar = 5 cm. Enlarged images corresponding to each plant are linked with white dashed lines. **(B)** TRV-mediated expression of VdrsR-1 slowed plant growth and delayed floral transition. Photographs were taken at 14 dpi. Scale bar = 5 cm. **(C)** The accumulation levels of VdrsR-1 and miR157d were detected by Northern blotting. U6 hybridization served as loading control. Northern blotting signals were quantified and normalized first to those of U6 and then to TRV-infected plants. The experiments were repeated three times with similar results. **(D)** The relative expression level of endogenous *SPL13A*. The asterisk indicates significantly different expression of TRV-VdrsR-1-infected plants versus TRV-infected plants (*n* = 9; *t*-test, *P* < 0.05).

Compared to healthy growing mock-inoculated *Arabidopsis* plants, *V. dahliae*-infected plants displayed wilting and necrosis symptoms at 14 dpi (Figure 6A). At this time point, *V. dahliae*-infected plants showed serious stunting, while the main inflorescences of the mock plants started growing, which is associated with shoot maturation during the reproductive phase (Shikata et al., 2009). At 28 dpi, the main inflorescences were observed in a small number of *V. dahliae*-infected plants, while the mock plants all flowered (Figure 6A). The results showed that *V. dahliae* infection delayed the vegetative phase change and floral transition.

To determine whether VdrsR-1 contributes to morphological defects, we used a TRV vector to express VdrsR-1 in *Arabidopsis*. Expression of VdrsR-1 was detected in plants infected with TRV-VdrsR-1 at 14 dpi but not in those infected with the TRV control (Figure 6C). Consistent with VdrsR-1 expression, increased miR157d and decreased *SPL13A* but not other *SPL* genes were detected in TRV-VdrsR-1-infected plants compared with TRV-infected plants (Figures 6C,D and Supplementary Figure 8). Evidently, TRV-VdrsR-1-infected plants exhibited stunting and delayed floral transition compared to TRV-infected plants (Figure 6B and Supplementary Figure 9). Taken together, we concluded that *V. dahliae* caused plant stunting and delayed floral transition, at least in part attributed to the fungal trans-kingdom VdrsR-1 promoting plant miR157d accumulation.

## Discussion

Bidirectional transmission of RNAi signals plays important roles in host-pathogen interactions. On the one hand, plant hosts export specific endogenous miRNAs into pathogens to inhibit their invasion by targeting virulence genes (Wang et al., 2016; Zhang et al., 2016). On the other hand, pathogens deliver sRNAs into host cells, which may function as RNA effectors to facilitate their colonization by interfering with host immunity (Weiberg et al., 2013; Dunker et al., 2020; Ji et al., 2021). In this study, we disclosed a new mechanism for the action of a fungal RNA effector.

By immunoprecipitation with AGO1 from *V. dahliae*-infected *Arabidopsis* plants, we obtained a number of *V. dahliae* sRNAs that were most likely associated with the host AGO1 protein and assumed to be trans-kingdom VdsRNAs (Figures 1C,D), which possibly matched host putative target genes in extensive pathways, including immune system processing and developmental processes (Figure 2). Among these, a 24-nt sRNA derived from *V. dahliae* rRNA, VdrsR-1, was shown to be a real trans-kingdom sRNA loaded into plant AGO1 and targeted to the host *MIR157d* (Figures 3A,C). A large number and various lengths of rRNA-derived sRNAs were generated in *V. dahliae* (Figure 3B). Whether *V. dahliae* rRNA-derived sRNAs play roles in the rDNA damage response, similar to the function of rDNA locus-derived sRNAs in the classical fungus *Neurospora* (Lee et al., 2009), requires further investigation. Interestingly, despite the large number and various lengths, only the 24-nt *V. dahliae* rRNA-derived sRNA VdrsR-1 was detected in AGO1-IP samples, hinting at the existence of a selective mechanism for either the delivery of VdsRNAs into plant cells or the loading of VdsRNAs into AGO1.

*Arabidopsis* AGO1 is the key factor mediating target mRNA cleavage, normally at the miRNA complementary site between nucleotide positions 10 and 11 of the miRNAs. Although unable to detect cleavage by using 5’ RACE, the perfect match of the first 14 nucleotides between VdrsR-1 and *MIR157d* suggests that AGO1-loaded VdrsR-1 possibly mediates cleavage of *MIR157d*. The detection of reduced *MIR157d* with increased accumulation of miR157d (Figures 4A,B) and a possible miR157d-containing intermediate (Supplementary Figure 1B) in *V. dahliae*-infected plants suggests that the AGO1-VdrsR-1-mediated action facilitated DCL1 processing on *MIR157d*, which bears a typical stem-loop structure with a large terminal loop. Indeed, complex secondary structures are the determinants for DCL1 processing (Bologna et al., 2009, 2013; Liu et al., 2012; Zhu et al., 2013). The biogenesis mechanism of miRNAs in *Arabidopsis* has been well analyzed in detail (Yu et al., 2017; Song et al., 2019). miRNA biogenesis begins with the cleavage of the terminal loop and then with additional cleavage by DCL1 until mature miRNAs are released (Bologna et al., 2009). Generally, the DCL1-mediated process of miRNAs is completed within the nucleus, whereas AGO1-directed cleavage of miRNA target occurs within the cytoplasm (Yu et al., 2017; Song et al., 2019; Wang et al., 2019). However, AGO1 has also been found to localize and function in nucleus (Song et al., 2019). The pairing of VdrsR-1 to the junction region of the terminal loop and the upper stem of *MIR157d* (Supplementary Figure 4) might guide AGO1-mediated removal of the terminal loop, likelihood undertaking the first cut of DCL1 in *MIR157d*, leading to facilitation of the subsequent cuts of DCL1 on the *MIR157d* stem-loop. An alternative possible mechanism of the effect of AGO1/VdrsR-1 on increasing the accumulation of miR157d might resemble the biosynthesis process of phased secondary siRNAs (phasiRNAs), a special class of siRNAs which the production requires AGO1/7-miRNA-directed cleavage of the target mRNA, and subsequently processed into phasiRNAs by a given DCL protein (Fei et al., 2013; Liu et al., 2020). Nevertheless, the in-depth mechanism of how this 24-nt trans-kingdom VdrsR-1 increased miR157d expression requires further investigation.

Previous studies reported that the miR156/157 family regulates vegetative and floral transitions by targeting *SPL* genes in several plant species (Schwab et al., 2005; Wu and Poethig, 2006; Chuck et al., 2007; Gandikota et al., 2007; Shikata et al., 2009, 2012; Xu et al., 2016; Liu et al., 2017; Gao et al., 2018; He X. et al., 2018). Induction of miR157d and miR157a/b/c upon *V. dahliae* infection was detected by Northern blotting and in AGO1-IPed libraries. However, only the transcripts of two *SPL* genes, *SPL13A/B*, were notably reduced, suggesting that the increased miR157a/b/c upon *V. dahliae* infection had little effect on the regulation of other *SPL* genes in leaves of the adult phase. Indeed, a previous study showed that *SPL* transcripts were differentially responsive to miR156/miR157, and most family members play roles in juvenile leaves (He J. et al., 2018). Moreover, in addition to developing wilting and necrosis symptoms, *V. dahliae*-infected *Arabidopsis* plants exhibited delayed floral transition and late flowering (Figure 6A). These phenotypic changes were at least in part attributed to the trans-kingdom VdrsR-1, as TRV-expressed VdrsR-1 in *Arabidopsis* plants also exhibited delayed floral transition and late flowering (Figure 6B), accompanied by increased miR157d and decreased *SPL13A/B* (Figures 6C,D). The predicted target of the fungal trans-kingdom VdrsR-1 was only the precursor of miR157d, *MIR157d*, but not other family members. The results from transient expression assays also demonstrated that VdrsR-1-promoted miR157d accumulation was not due to the miR157d mature sequence but the precursor *MIR157d*, which bears a typical secondary structure (Figure 5 and Supplementary Figure 6). *SPL13A/B* transcripts were very slightly increased in miR156a/c/d miR157a/c mutant plants (He J. et al., 2018), whereas, only *SPL13A/B* but not other *SPL* transcripts was significantly reduced in *V. dahliae*-infected (Figure 4C and Supplementary Figure 2) and VdrsR-1-expressing plants (Figure 6D and Supplementary Figure 8). Therefore, we reasoned that the delayed floral transition was mainly due to the increased accumulation of miR157d which targeted *SPL13A/B* transcripts.

In light of the arms race between the host plant and *V. dahliae*, which is a hemibiotrophic pathogenic fungus, extending the vegetative growth stage of host plants would benefit fungal propagation during the biotrophic life cycle. Therefore, in addition to delivery sRNAs functioning as RNA effectors to facilitate fungal colonization by interfering with host immunity (Axtell and Bowman, 2008; Weiberg et al., 2013; Dunker et al., 2020; Ji et al., 2021), we disclosed a novel strategy for a trans-kingdom sRNA of *V. dahliae* by secreting VdrsR-1 into plant cells to employ the host miR157d/*SPL13A/B*, a phase transition regulatory module, to prolong vegetative growth for better feeding on living plant tissues.

## Data Availability Statement

The data presented in the study are available in the National Center for Biotechnology Information (NCBI) repository under accession number PRJNA794992.

## Author Contributions

H-SG and J-HZ conceived the study, designed the research, wrote the manuscript, and discussed the results. J-HZ analyzed the sequencing data. B-SZ and Y-CL performed the sampling and molecular work. All authors commented on the manuscript.

## Conflict of Interest

The authors declare that the research was conducted in the absence of any commercial or financial relationships that could be construed as a potential conflict of interest.

## Publisher’s Note

All claims expressed in this article are solely those of the authors and do not necessarily represent those of their affiliated organizations, or those of the publisher, the editors and the reviewers. Any product that may be evaluated in this article, or claim that may be made by its manufacturer, is not guaranteed or endorsed by the publisher.

## Footnotes

1
https://www.omicshare.com/

## Supplementary Material

Supplementary Figure 1 The accumulation of miR157d in Mock and *V. dahliae*-infected plants. **(A)** Heatmap showing the log2 transformations of the fold-change (Infected vs. Mock) of the miR156/157 family members. The values are labeled on the squares and the clustering results are based on the mean values of three biological replicates. **(B)** An overexposed Northern blotting membrane of detection for the accumulation of miR157d. Loading slots are indicated. Arrows show the possible *MIR157d* precursor (upper band) and miR157d-containing intermediate (lower band).

Supplementary Figure 2 The relative expression level of endogenous *SPL* genes. The asterisk indicates significantly different expression of infected plants versus mock plants (*n* = 9; *t*-test, *P* < 0.05).

Supplementary Figure 3 Alignment of *SPL13A* and *SPL13B* sequences. The miR157d targeted region is marked by a red line, and the probes used for Northern blotting are marked by black lines.

Supplementary Figure 4 The predicted secondary structure of *MIR157d*. The mature miR157d and VdrsR-1 sequences are presented in red and blue letters, respectively.

Supplementary Figure 5 Various miRNA precursors were used to test for the production of 24-nt VdrsR-1.

Supplementary Figure 6 The predicted secondary structure of *MIR157dm*. The mature miR157d and VdrsR-1 sequences are presented in red and blue letters, respectively. The green dashed lines mark the mutated nucleotides within the *MIR157d* and *MIR157dm* sequences.

Supplementary Figure 7 The predicted secondary structure of *MIR159a*. The artificial miRNA sequences targeting *MIR159a* are presented in blue letters. The mature miR159a sequence are presented in red letters.

Supplementary Figure 8 The relative expression level of endogenous *SPL* genes in TRV-, and TRV-VdrsR-1-infected plants. The asterisks indicate significantly different expression (*n* = 9; *t*-test, *P* < 0.05).

Supplementary Figure 9 The numbers of flowering plants after TRV and TRV-VdrsR-1 infection, respectively. The number of total plant was shown above the column. The asterisk indicates that the two groups have significantly different (Chi-square test, *P* < 0.05).
