# Supplementary material for: Verticillium dahliae Secretes Small RNA to Target Host MIR157d and Retard Plant Floral Transition During Infection
Source: Front Plant Sci. 2022 Apr 18;13:847086. doi: 10.3389/fpls.2022.847086 (PMC9062233; doi:10.3389/fpls.2022.847086)
Supplement: Supplementary file 1 [file Table_1.DOCX]

**Table S1. Oligonucleotide sequences were used in this study.**

| **Primer name** | **Primer sequence (5’ to 3’)** | **Purpose** |
| --- | --- | --- |
| qSPL10-F | GCAAGCCACAAGGAGTATTT | qPCR |
| qSPL10-R | GAGGGTGCTACCAGTAAACA | qPCR |
| qSPL2-F | GGAGAATGCTAAAGGTTTGGGACT | qPCR |
| qSPL2-R | AACCGACGCTCTACGCCACT | qPCR |
| qSPL15-F | CTCTATTCTCCTCCGTCCTTAC | qPCR |
| qSPL15-R | GACCAAACCTGAGTCCACCA | qPCR |
| qSPL9-F | GCAGGTTTCATCAGCTTCCG | qPCR |
| qSPL9-R | CCGCCTCATCACTCTTGTAT | qPCR |
| qSPL11-F | GCCAACAGTGTAGCAGGTTC | qPCR |
| qSPL11-R | TGTCTAGGCTTTGTATCATAAGTG | qPCR |
| qSPL6-F | GTCAAGTTTATGGGTGTAGTAAG | qPCR |
| qSPL6-R | GTAGGAAATAGAATGCAGGTT | qPCR |
| qSPL13A/B-F | TTCTGTCATCCTCCTCGTCG | qPCR |
| qSPL13A/B-F | AATGAAACGGGAATGTCTGC | qPCR |
| qmi157-F | GTGGAGGGTGATAGTGTGGTTGCT | qPCR |
| qmi157-R | GTAGAGAGAAATAGAGAAAGAAAG | qPCR |
| qmi159-F | GTAGAGCTCCTTAAAGTTCAAACA | qPCR |
| qmi159-R | GTAGAGCTCCCTTCAATCCAAAGA | qPCR |
| qSPL13A-F | TTGCATTATTGAAGTAGCAG | qPCR |
| qSPL13A-R | CTTACCAAATCCATACACCT | qPCR |
| At_33380-F | AGGAGAGGAAGAGCCTGAGGA | qPCR |
| At_33380-R | ATCTCACTGCAGCACCACCA | qPCR |
| qSPL13B-F | CTTCTGTCATCCTCCTCGTCG | qPCR |
| qSPL13B-R | AATGTCTGCGGAAGACGGAC | qPCR |
| NBGAPDH-F | AGCTCAAGGGAATTCTCGATG | qPCR |
| NBGAPDH-R | AACCTTAACCATGTCATCTCCC | qPCR |
| mi168reverse | GTCGTATCCAGTGCAGGGTCCGAGGTATTCGCACTGGATACGACTTCCCG | RT-PCR |
| mi168-F | GCGTCGCTTGGTGCAGGT | RT-PCR |
| mi168-R | AGTGCAGGGTCCGAGGTAT | RT-PCR |
| mi162reverse | GTCGTATCCAGTGCAGGGTCCGAGGTATTCGCACTGGATACGACCTGGAT | RT-PCR |
| mi162-F | CGCGTCGATAAACCTCTGC | RT-PCR |
| mi162-R | AGTGCAGGGTCCGAGGTATT | RT-PCR |
| siRNA1003reverse | GTCGTATCCAGTGCAGGGTCCGAGGTATTCGCACTGGATACGACATGCCA | RT-PCR |
| siRNA1003-F | GCGGCGAGACCGTGAGGCCAA | RT-PCR |
| siRNA1003-R | AGTGCAGGGTCCGAGGTATT | RT-PCR |
| VdmilR-1reverse | GTCGTATCCAGTGCAGGGTCCGAGGTATTCGCACTGGATACGACTAACCA | RT-PCR |
| VdmilR-1-F | GCGCGTTCCGATTAGTCTAG | RT-PCR |
| VdmilR-1-R | AGTGCAGGGTCCGAGGTATT | RT-PCR |
| VdrsR-1Reverse | GTCGTATCCAGTGCAGGGTCCGAGGTATTCGCACTGGATACGACTCGGGT | RT-PCR |
| VdrsR-1-F | GCGGAGAGCATATAGGGTTGG | RT-PCR |
| VdrsR-1-R | AGTGCAGGGTCCGAGGTATT | RT-PCR |
| miR157dprobe | AGTGCTCTCTATCTTCTGTCA | Northern blot |
| miR159aprobe | TAGAGCTCCCTTCAATCCAAA | Northern blot |
| VdrsR-1probe | TCGGGTCCAACCCTATATGCTCTC | Northern blot |
| MIR157asr-1probe | GTGCTCTCAGATGAACTACT | Northern blot |
| U6-probe | CTCGATTTATGCGTGTCATCCTTGC | Northern blot |
| Dicer1probe1-F | TAGGGATCGATAGACCGTGG | Northern blot |
| Dicer1probe1-R | GACGCCGTTTTCATCAGTGG | Northern blot |
| Dicer1probe2-F | AGGGATCGATAGACCGTGGA | Northern blot |
| Dicer1probe2-R | CGCCATTCTTTTGCAACCCA | Northern blot |
| Dicer1probe3-F | GGGATCGATAGACCGTGGAA | Northern blot |
| Dicer1probe3-R | ACCGCCATTCTTTTGCAACC | Northern blot |
| SPL13A/Bprobe-F | CATAATCGAAGACGACGGAAGC | Northern blot |
| SPL13A/Bprobe-R | GGGACTGACGACGAGGAGGA | Northern blot |
| MIR5653VdrsR-1-F | AACACGGGGGACTCTAGAACGGGTAACCCTTAAACTCTCTCA | 35S-MIR5653_VdrsR-1_ construction |
| MIR5653VdrsR-1-R | TCGGGGAAATTCGAGCTCTCGGGTCCAACCCTATATGCTCTC | 35S-MIR5653_VdrsR-1_ construction |
| MIR5653T159-F | AACACGGGGGACTCTAGATCTTGCTTTAATGCAATTACTTCA | 35S-amiR_t159_ construction |
| MIR5653T159-R | TCGGGGAAATTCGAGCTCACTTGCCCTTTAATGGCTTTTACT | 35S-amiR_t159_ construction |
| 35SMIR157d-F | AACACGGGGGACTCTAGAGTGGAGGGTGATAGTGTGGTTGCT | 35S-MIR157d construction |
| 35SMIR157d-R | TCGGGGAAATTCGAGCTCGTAGAGAGAAATAGAGAAAGAAAG | 35S-MIR157d construction |
| 35SMIR159a-F | AACACGGGGGACTCTAGAGTAGAGCTCCTTAAAGTTCAAACA | 35S-MIR159a construction |
| 35SMIR159a-R | TCGGGGAAATTCGAGCTCGTAGAGCTCCCTTCAATCCAAAGAAGAG | 35S-MIR159a construction |
| 35SMIR157dm-F | AACACGGGGGACTCTAGAGTGGAGGGTGATAGTGTGGTTGCT | 35S-MIR157dm construction |
| 35SMIR157dm-R | TCGGGGAAATTCGAGCTCGTAGAGAGAAATAGAGAAAGAAAG | 35S-MIR157dm construction |
| 35SMIR157dasr-F | AACACGGGGGACTCTAGAGTGGAGGGTGATAGTGTGGTTGC | 35S-MIR157d_asr-1_ construction |
| 35SMIR157dasr-R | TCGGGGAAATTCGAGCTCGTAGAGAGAAATAGAGAAAGAA | 35S-MIR157d_asr-1_ construction |
| 35SSPL13B-F | AACACGGGGGACTCTAGACCTTTCTTCCCATTTTGTGGGATC | 35S-AtSPL13B construction |
| 35SSPL13B-R | TCGGGGAAATTCGAGCTCCTTAAAAAGAGTTACAAAAGTATA | 35S-AtSPL13B construction |
| TRV-VdrsR-1-F | GGTTACCGAATTCTCTAGAACGGGTAACCCTTAAACTCTCTCA | TRV-MIR5653_VdrsR-1_ construction |
| TRV-VdrsR-1-R | TCGAGACGCGTGAGCTCTCGGGTCCAACCCTATATGCTCTC | TRV-MIR5653_VdrsR-1_ construction |
