# Supplementary figures and images for: Verticillium dahliae Secretes Small RNA to Target Host MIR157d and Retard Plant Floral Transition During Infection
Source: Front Plant Sci. 2022 Apr 18;13:847086. doi: 10.3389/fpls.2022.847086 (PMC9062233; doi:10.3389/fpls.2022.847086)

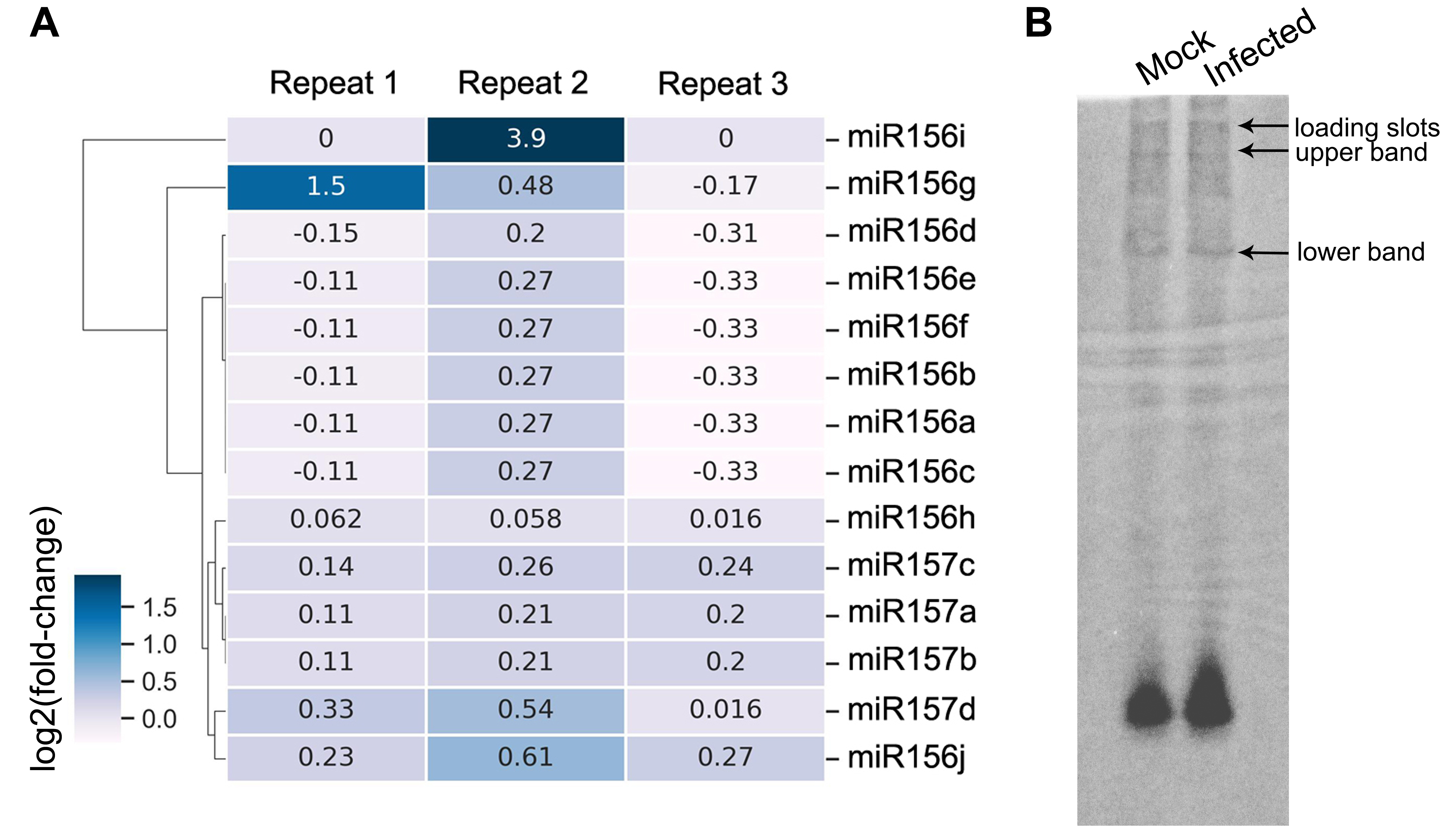

Supplement: Supplementary Figure 1 — The accumulation of miR157d in Mock and V. dahliae-infected plants. (A) Heatmap showing the log2 transformations of the fold-change (Infected vs. Mock) of the miR156/157 family members. The values are labeled on the squares and the clustering results are based on the mean values of three biological replicates. (B) An overexposed Northern blotting membrane of detection for the accumulation of miR157d. Loading slots are indicated. Arrows show the possible MIR157d precursor (upper band) and miR157d-containing intermediate (lower band). [file Image_1.JPEG]

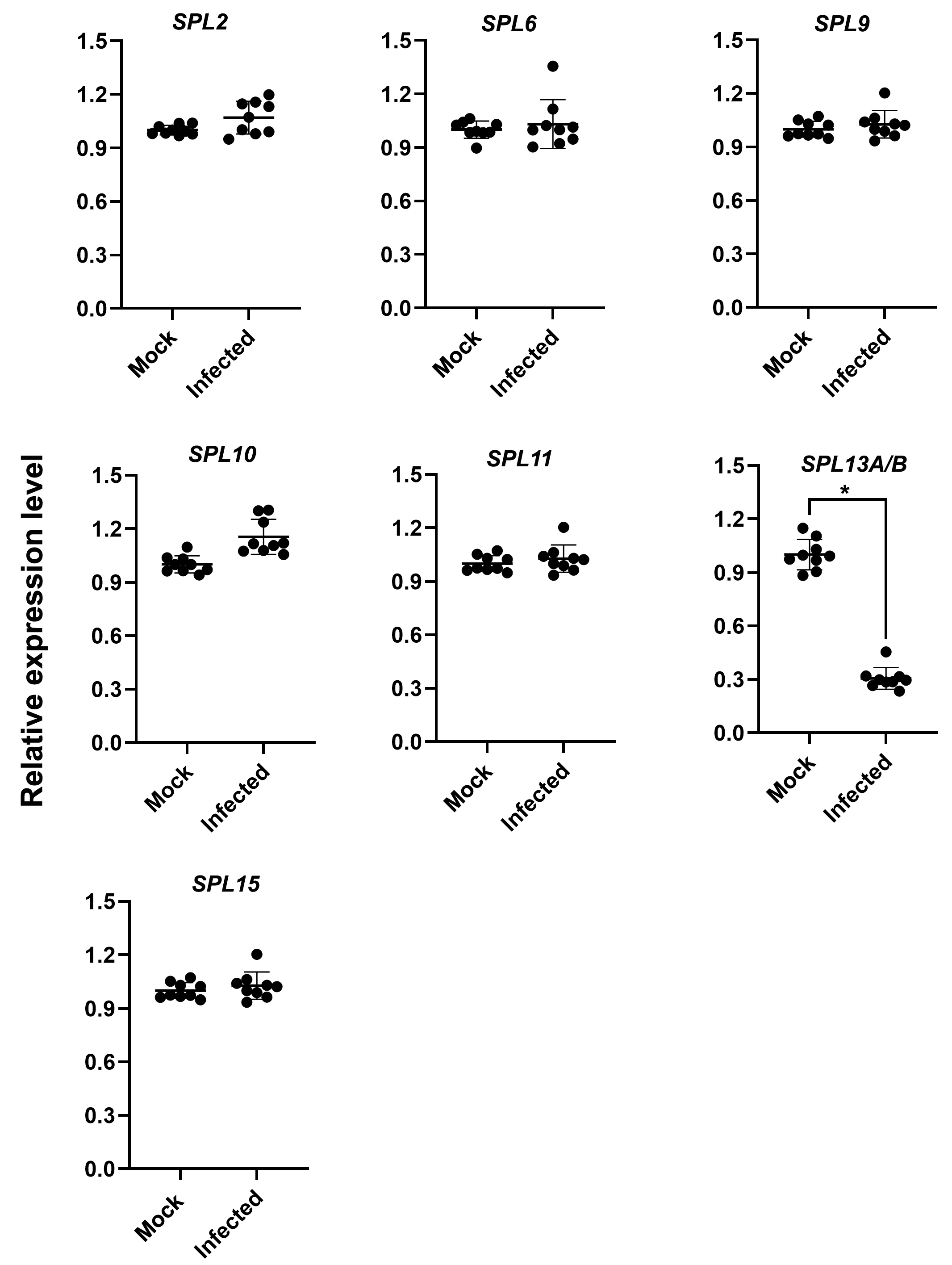

Supplement: Supplementary Figure 2 — The relative expression level of endogenous SPL genes. The asterisk indicates significantly different expression of infected plants versus mock plants (n = 9; t-test, P < 0.05). [file Image_2.JPEG]

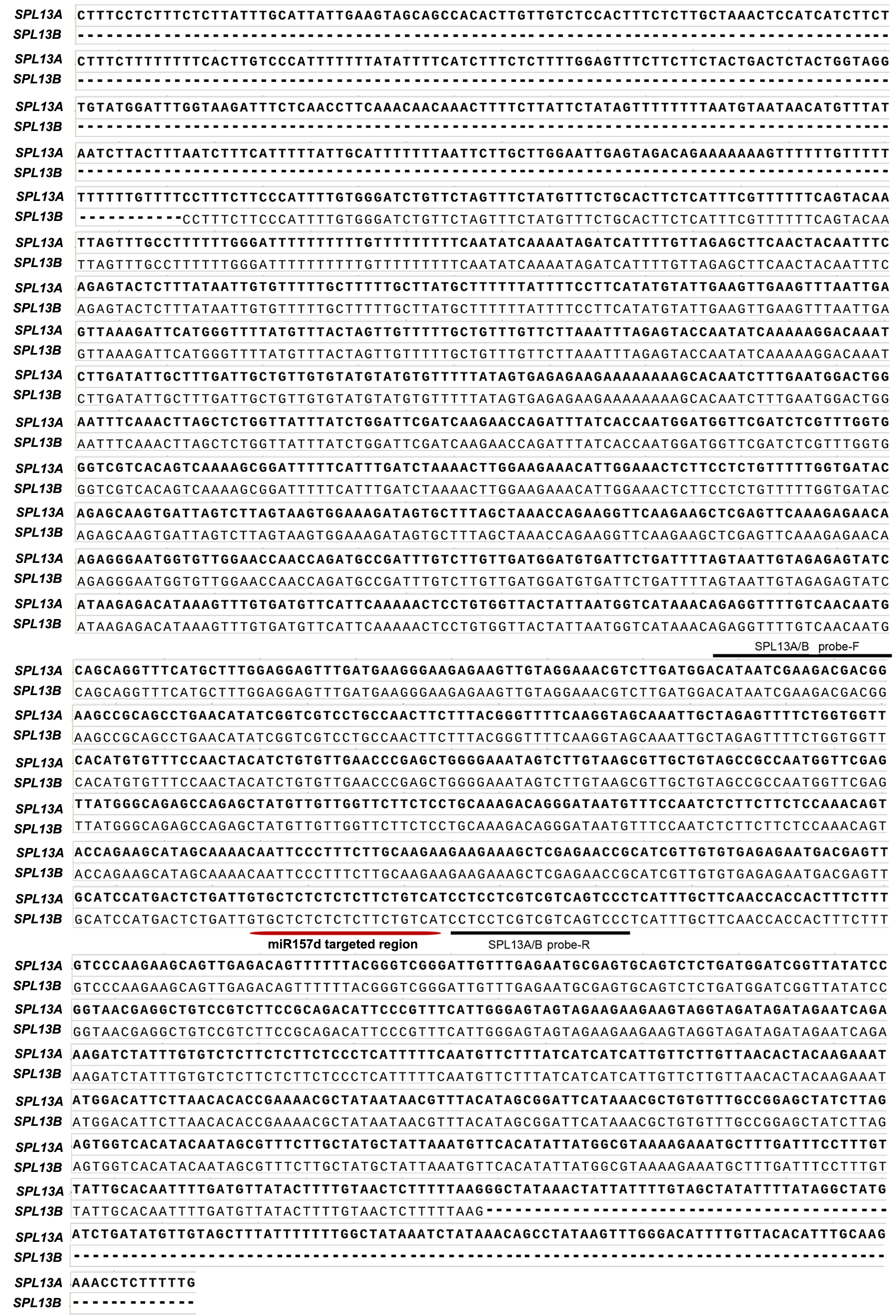

Supplement: Supplementary Figure 3 — Alignment of SPL13A and SPL13B sequences. The miR157d targeted region is marked by a red line, and the probes used for Northern blotting are marked by black lines. [file Image_3.JPEG]

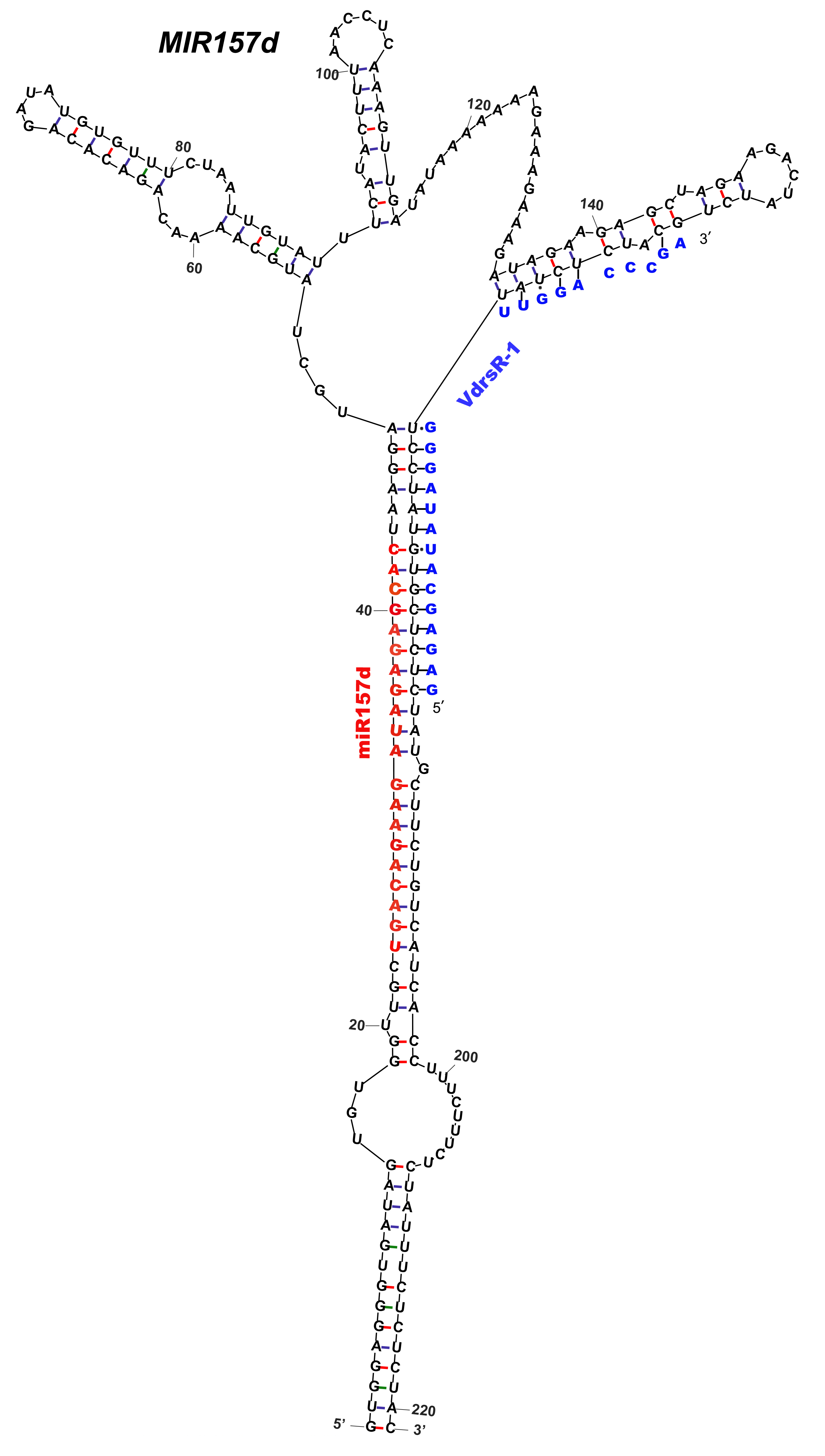

Supplement: Supplementary Figure 4 — The predicted secondary structure of MIR157d. The mature miR157d and VdrsR-1 sequences are presented in red and blue letters, respectively. [file Image_4.JPEG]

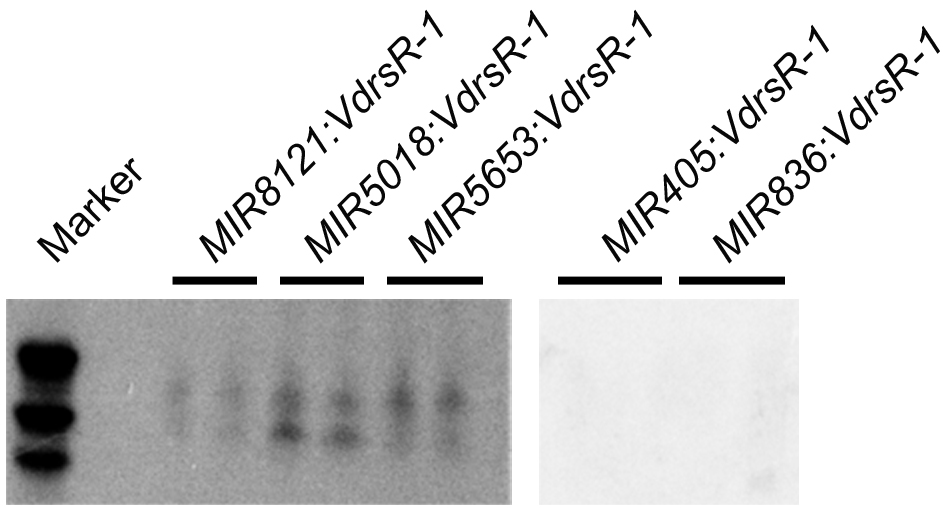

Supplement: Supplementary Figure 5 — Various miRNA precursors were used to test for the production of 24-nt VdrsR-1. [file Image_5.JPEG]

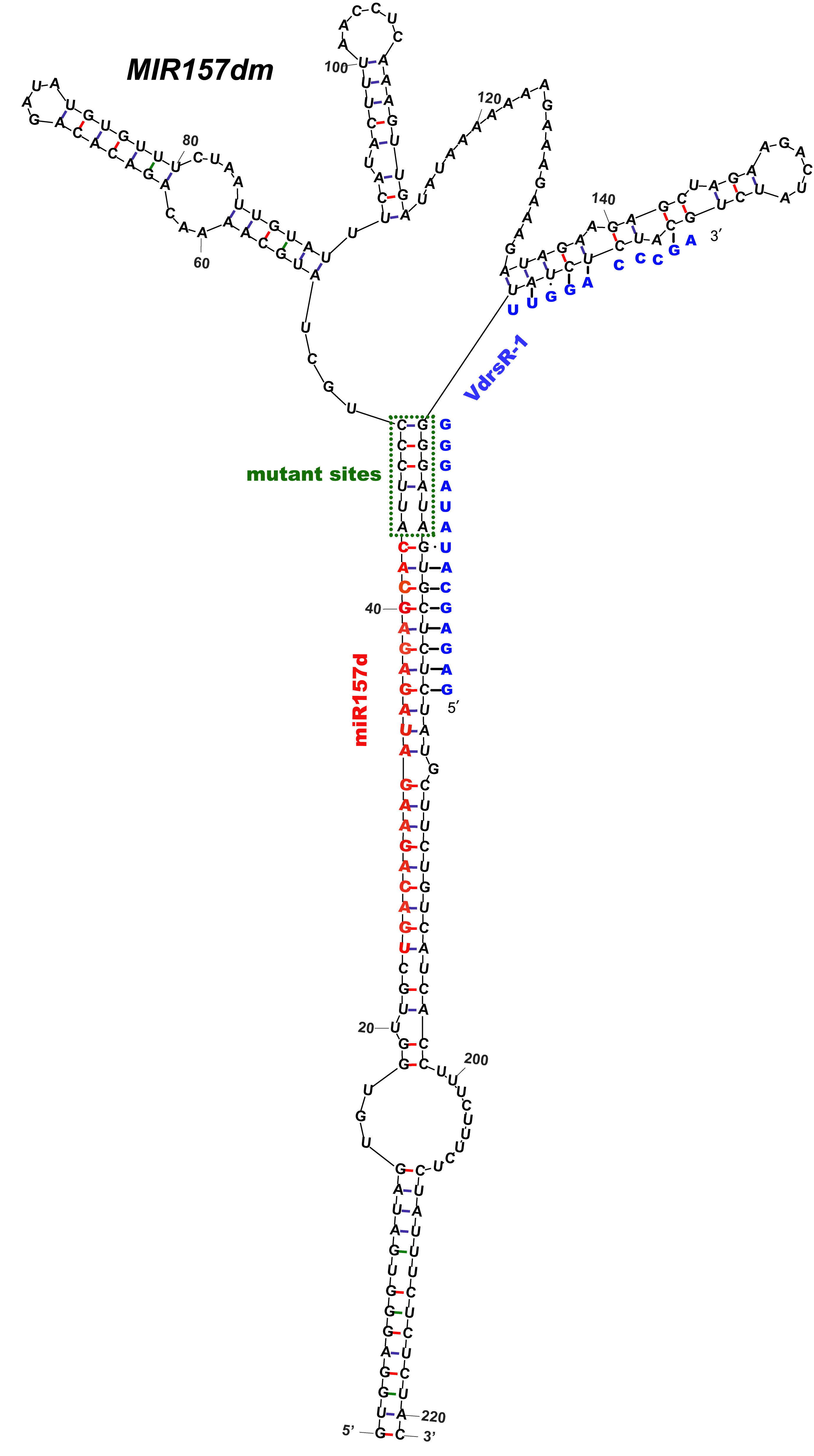

Supplement: Supplementary Figure 6 — The predicted secondary structure of MIR157dm. The mature miR157d and VdrsR-1 sequences are presented in red and blue letters, respectively. The green dashed lines mark the mutated nucleotides within the MIR157d and MIR157dm sequences. [file Image_6.JPEG]

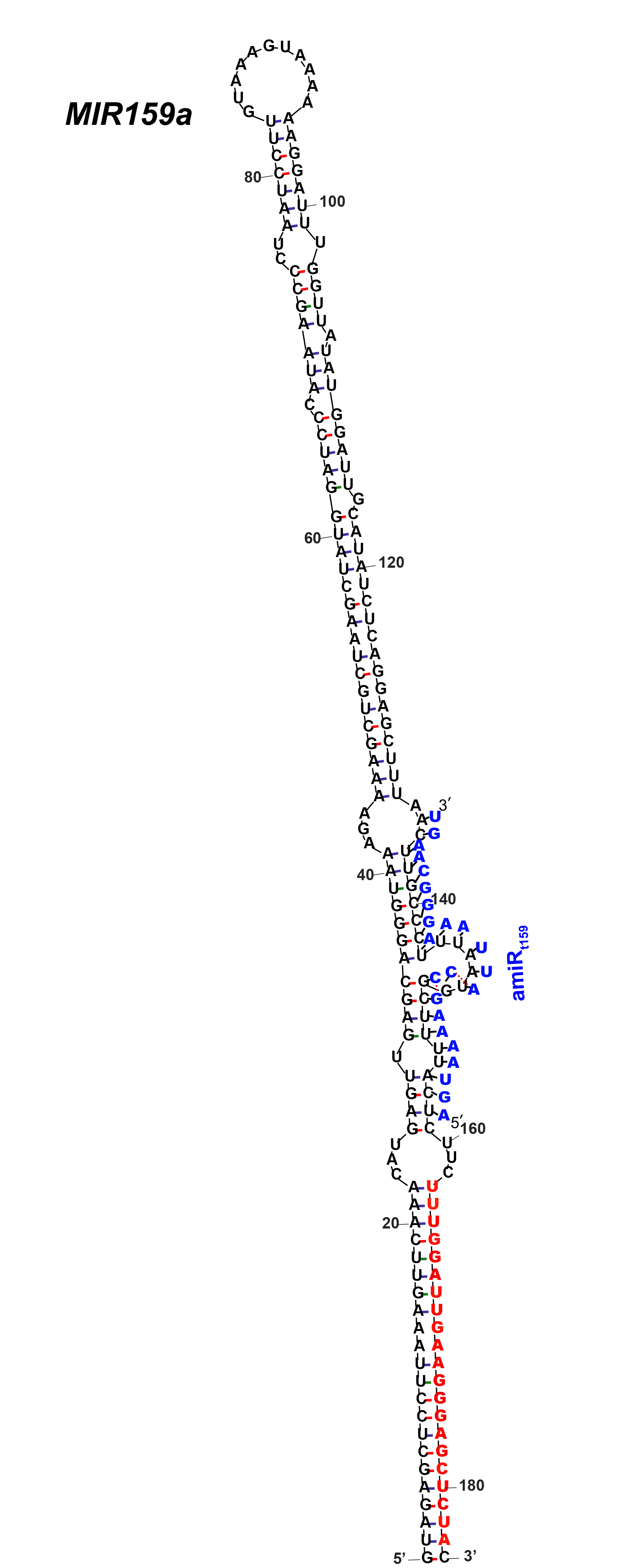

Supplement: Supplementary Figure 7 — The predicted secondary structure of MIR159a. The artificial miRNA sequences targeting MIR159a are presented in blue letters. The mature miR159a sequence are presented in red letters. [file Image_7.JPEG]

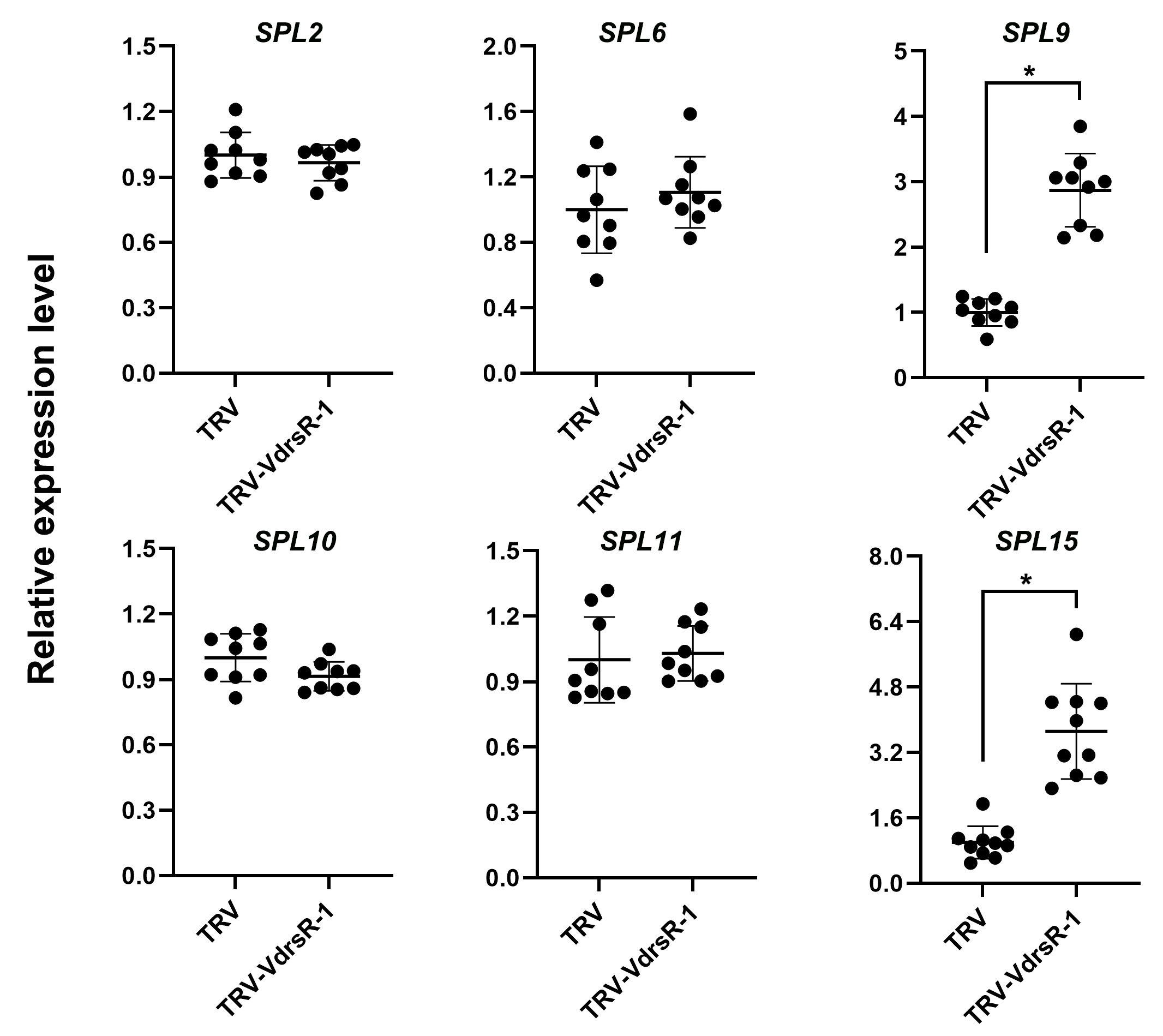

Supplement: Supplementary Figure 8 — The relative expression level of endogenous SPL genes in TRV-, and TRV-VdrsR-1-infected plants. The asterisks indicate significantly different expression (n = 9; t-test, P < 0.05). [file Image_8.JPEG]

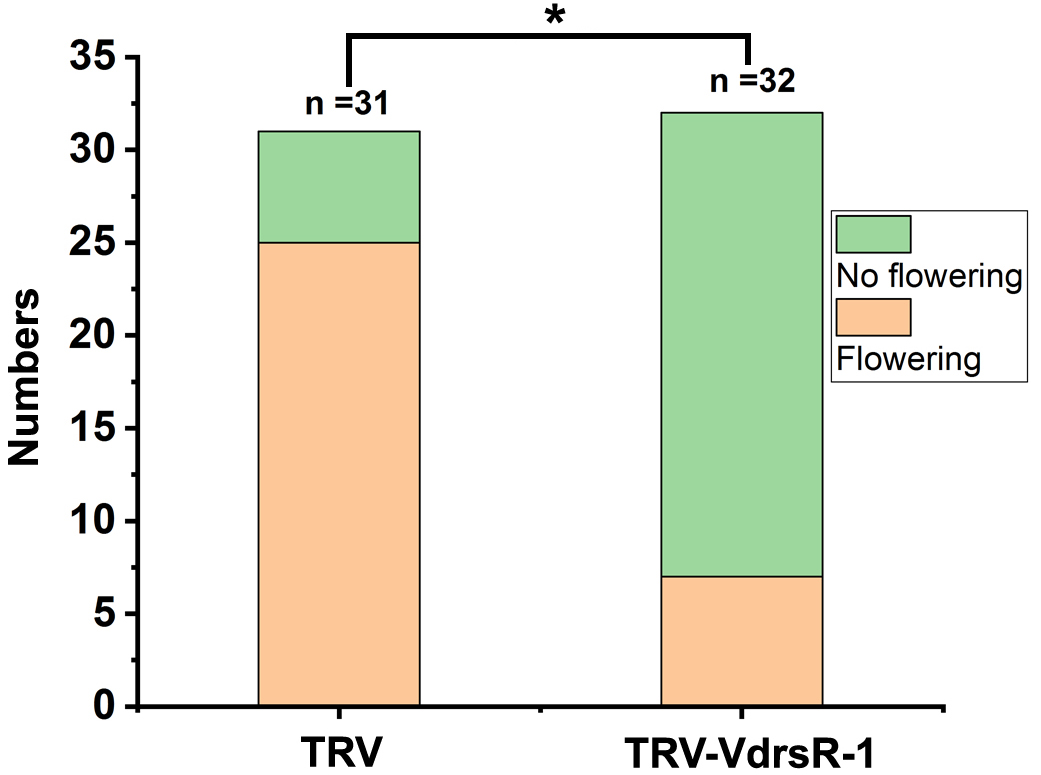

Supplement: Supplementary Figure 9 — The numbers of flowering plants after TRV and TRV-VdrsR-1 infection, respectively. The number of total plant was shown above the column. The asterisk indicates that the two groups have significantly different (Chi-square test, P < 0.05). [file Image_9.jpg]
